# Supplementary material for: Correlation between Overconfidence and Learning Motivation in Postgraduate Infection Prevention and Control Training
Source: Int J Environ Res Public Health. 2022 May 9;19(9):5763. doi: 10.3390/ijerph19095763 (PMC9100152; doi:10.3390/ijerph19095763)
Supplement: Supplementary file 1 [file ijerph-19-05763-s001.zip › Supplementary material B_eng_BG.pdf]

Supplementary Materials B
Relevant items of the questionnaire for the present study

Table S1. Demographic items in the questionnaire in German and English

German	English
Zuerst einige Fragen an Sie ...	Firstly, we have some questions for you…
Welches Geschlecht haben Sie?<br>-weiblich,<br>-männlich<br>-divers/transgender/transidentical<br>-keine Angabe	Which gender are you?<br>-female<br>-male<br>-diverse/transgender/transidentical<br>-Not applicable
Wie alt Sie sind?<br>-möchten Sie Ihr Alter nicht angeben, tragen Sie bitte "0" ein	How old are you?<br>-If you do not want to disclose your age, you can enter “0”
Wie alt fühlen Sie sich?<br>-möchten Sie Ihr Alter nicht angeben, tragen Sie bitte "0" ein	How old do you feel?<br>-If you do not want to disclose your age, you can enter “0”
Zu welcher Profession gehören Sie?<br>-Gesundheits- und Krankenpflegekraft<br>-Ärztin / Arzt<br>-Rettungsdienstfachpersonal (RS,RA, NotSan)<br>-Andere<br>-keine Angabe	To which profession do you belong?<br>-nurse<br>-physician<br>-paramedic<br>-other<br>-not applicable
Wie ist ihr Ausbildungsstand?<br>-Ich bin Schülerin/Schüler oder Studierende/Studienerender<br>-Ich habe meine Ausbildung abgeschlossen<br>-Ich bin selbst Ausbilderin / Ausbilder<br>-keine Angabe	What is your level of education?<br>-Student/Trainee<br>-I have completed my professional training<br>-I am an instructor myself<br>-not applicable

Table S2. The relevant Items for the present study in the questionnaire in German and English

German	English
Wenn Sie an einer Schulung zur Händehygiene und Infektionsprävention teilnehmen, in wie weit treffen folgende Aussagen auf Sie zu?	When you think about hand hygiene training, to what extent does the following apply to you?
1.Ich glaube diese Schulung ist für mich interessant	I think that the training is interesting
2.Ich mache diese Schulung zu meinem eigenen Nutzen	I am doing this training for my own benefit
3.Ich mache diese Schulung, weil ich es muss	I am doing this training because I have to
4.Es mag gute Gründe geben, die Schulung zu machen, ich sehe für mich aber keine	There may be good reasons to do this training, but I don’t see any for myself
5.Ich glaube eine solche Schulung ist angenehm	I think that this training is pleasant
6.Ich denke eine solche Schulung ist gut für mich	I think that such a training is good for me
7.Ich mache es, weil es etwas ist, was ich tun muss	I do it because it is something that I have to do
8.Ich mache es, denke aber nicht, dass es die Zeit wert ist	I do it but do not think that it is worth the time
9.Ich mache es, weil es Spaß macht	I do it because it is fun
10.Ich nehme teil, weil es meine eigene Entscheidung ist	I participate because it is my personal decision
11.Ich nehme teil weil ich keine andere Wahl habe	I participate because I don’t have another choice
12.Ich weiß nicht, aber ich sehe nicht, was mir die Schulung bringen soll	I don’t know; I don’t see what this training brings me
13.Ich fühle mich gut, wenn ich die Schulung besuche	I feel good when attending this training
14.Ich glaube die Schulung ist wichtig für mich	I believe that this training is important for me
15.Ich fühle, dass ich an der Schulung teilnehmen muss	I feel that I have to participate in this training
16.Ich nehme teil, bin mir aber unsicher, ob es gut es so etwas zu verfolgen	I participate, but I am unsure whether it is good to pursue it
17.Fragen zur Selbsteinschätzung	Questions on Self-assessment
18.Ich führe die hygienische Händedesinfektion situationsgerecht durch.	I carry out hand hygiene if indicated in a situation
19.Ich erkenne die Indikationen für eine hygienische Händedesinfektion	I identify the indications for hand hygiene
20.Ich wähle das benötigte Händedesinfektionsmittel situationsgerecht aus	I choose the appropriate disinfection agent for hand hygiene when indicated

21.Ich erkenne Fehler in der Durchführung der hygienischen Händedesinfektion bei anderen Personen	I identify mistakes in the conduction of hand hygiene in other persons
22.Ich greife korrigierend ein, wenn ich einen Fehler in der Durchführung der hygienischen Händedesinfektion bemerke	I correct others readily, if I notice a mistake in hand hygiene
23.Ich nehme situationsgerecht Rückmeldungen an, wenn ich durch eine andere Person auf einen Fehler in der hygienischen Händedesinfektion hingewiesen werde	I accept feedback if others correct me for an error in hand hygiene if appropriate to the situation
24.Meine Kollegen setzen die hygienische Händedesinfektion situationsgerecht um	My colleagues carry out hand hygiene when indicated
25.Meine Kollegen wissen die jeweiligen Indikationen für eine hygienische Händedesinfektion	My colleagues know the indications for hand hygiene
26.Meine Kollegen wählen das notwendige Desinfektionsmittel für eine hygienische Händedesinfektion korrekt aus	My colleagues correctly choose the indicated disinfection agent
27.Meine Kollegen korrigieren mich situationsgerecht, wenn sie einen Fehler in meiner Händedesinfektion bemerken	My colleagues correct me appropriately if they notice a mistake in my hand hygiene
28.Meine Kollegen nehmen situationsgerecht Rückmeldungen und Hinweise an, wenn sie auf einen Hygienefehler angesprochen werden	My colleagues accept feedback according to the situation when they are corrected by others
29.Ärztinnen und Ärzte führen die hygienische Händedesinfektion situationsgerecht aus	Physicians carry out hand hygiene when indicated
30.Ärztinnen und Ärzte korrigieren mich situationsgerecht, wenn sie einen Fehler in meiner Händedesinfektion bemerken	Physicians correct me appropriately, if they notice a mistake in my hand hygiene
31.Ärztinnen und Ärzte nehmen situationsgerecht Rückmeldungen und Hinweise an, wenn sie auf einen Hygienefehler angesprochen werden	Physicians accept feedback according to the situation when they are corrected by others
32.Pflegekräfte führen die hygienische Händedesinfektion situationsgerecht aus	Nurses carry out hand hygiene when indicated
33.Pflegekräfte korrigieren mich situationsgerecht, wenn sie einen Fehler in meiner Händedesinfektion bemerken	Nurses correct me appropriately, if they notice a mistake in my hand hygiene

34.Pflegekräfte nehmen situationsgerecht Rückmeldungen und Hinweise an, wenn sie auf einen Hygienefehler angesprochen werden	Nurses accept feedback according to the situation when they are corrected by others
35.Angehörige des Rettungsdienstes führen die hygienische Händedesinfektion situationsgerecht aus	Paramedics carry out hand hygiene when indicated
36.Angehörige des Rettungsdienstes korrigieren mich situationsgerecht, wenn sie einen Fehler in meiner Händedesinfektion bemerken	Paramedics correct me appropriately, if they notice a mistake in my hand hygiene
37.Angehörige des Rettungsdienstes nehmen situationsgerecht Rückmeldungen und Hinweise an, wenn sie auf einen Hygienefehler angesprochen werden	Paramedics accept feedback according to the situation when they are corrected by others
Alle Items (1-37) waren auf 5 Likert Skala 1=trifft nicht zu bis 5=trifft voll zu	All Items (1-37) were scaled on 5 Likert Scale 1=do not agree till 5=totally agree
Der glaubwürdig maximale Schaden einer unterlassenen Händedesinfektion ist<br>Unbedeutend (1)<br>gering - aber keine nachhaltigen Schäden (2)<br>Spürbar- mit verlängertem Aufenthalt im Krankenhaus (3)<br>Kritisch - mit bleibenden körperlichen Schäden (4)<br>Katastrophal - mit Todesfolge (5)	The credible maximum damage of omitted hand hygiene is<br>insignificant (1),<br>minor-without lasting effects (2),<br>noticable- with a prolonged hospital stay (3),<br>critical -with lasting physical damages (4),<br>catastrophic- lethal (5)
Wie häufig kommt es vor, dass ein Patient den geschätzten Schaden durch eine ausgebliebene Händedesinfektion erleidet?<br>seltener als einmal in 3 Jahren (1),<br>häufiger als. einmal in 3 Jahren (2),<br>häufiger als einmal pro Jahr (3)<br>häufiger als einmal pro 3 Monate (4)<br>häufiger als einmal pro Monat(5)	How often does it occur that a patient incurs the estimated potential damage of omitted hand hygiene?<br>Uncommon (1x >3 years) (1),<br>seldom (once every 3 years) (2),<br>moderate (once a year)(3),<br>often (once every 3 months) (4),<br>very often (once a month) (5)
In folgenden Situationen setze ich die hygienische Händedesinfektion um Immer (>99%), Häufig (75-99%), Mittelmäßig (25-75%), Selten (1-25%), Niemals (< 1%)	I carry out hand hygiene in the following situations always (99%)(1), often (75–99%) (2), sometimes (25–75%) (3), seldomly (1-25%) (4), never (5)
Vor der Verbindung einer Infusionsleitung an eine periphere Venenverweilkanüle(WHO-2)	Before connecting an infusion to an IV line (WHO-2)

Nach dem Aufheben eines heruntergefallenen Handtuchs eines Patienten im Bad (WHO-5)	After picking up a patient’s used towel in the bathroom (WHO-5)
Vor dem Betreten eines Patientenzimmers bei einer Visite (no clear indication)	Before entering a patient's room during rounds (no clear indication)
Nach versehentlicher Kontamination der eigenen Hand mit Urin eines Patienten(WHO-3)	After accidental contamination of the own hand with patient’s urine (WHO-3)
Nach dem Vorbereiten eines Medikaments(no indication)	After preparing sterile IV medication (no indication)
Nach dem Abstellen eines benutzten Patientenbettes in einen Untersuchungsraum(WHO-5)	After parking a used patient’s bed in an examination room(WHO-5)
Vor der Begrüßung eines Patienten mit Handschlag(WHO-1)	Before greeting a patient with a handshake (WHO-1)
Nach dem Aufhelfen eines gestürzten Patienten(WHO-4)	After helping a patient stand up who has fallen(WHO-4)
Nach der Verabschiedung eines Patienten mit Handschlag(WHO-4)	After saying goodbye to a patient with a handshake(WHO-4)
Nach dem Essen in der Mittagspause (no clear indication)	After eating lunch in the canteen (no clear indication)
Vor dem Umstecken eines neuen Urin-Beutels an einen Harnblasendauerkatheter(WHO-2)	Before connecting a urinary catheter to a collection bag (WHO 2)
Vor dem Lagern eines Patienten im Bett oder auf einem OP-Tisch(WHO-1)	Before positioning a patient in their bed or on the operating table (WHO-1)
