# Supplementary material for: Correlation between Overconfidence and Learning Motivation in Postgraduate Infection Prevention and Control Training
Source: Int J Environ Res Public Health. 2022 May 9;19(9):5763. doi: 10.3390/ijerph19095763 (PMC9100152; doi:10.3390/ijerph19095763)
Supplement: Supplementary file 1 [file ijerph-19-05763-s001.zip › Supplementary materials A.pdf]

Supplementary materials A

Table S1

Curvilinear regression analysis of intrinsic motivation as criterion and overplacement as a predictor variable

	B	R^2	BCa 95% Lower	BCa 95% Upper
Constant	3.15		2.95	3.36
Overplacement	.10	.02	-.06	.28
Overplacement²-quadratic term	-.03	.02	-0.20	.21

Note. Dependent variable is intrinsic motivation, B-unstandardised regression coefficient; R^2-a proportion of the variance of the dependent variable explained by an independent variable; BCa 95% Lower and BCa 95% Upper-the borders of the 95% confidence interval after bootstrapping (Bias corrected and accelerated method) based on 1000 samples.

Table S2

Curvilinear regression analysis of identified regulation as criterion and overplacement as predictor variable

	B	R^2	BCa 95% Lower	BCa 95% Upper
Constant	3.79		3.56	4.01
Overplacement	.11	.01	-.08	.32
Overplacement ²-quadratic term	-.10	.06	-.27	.17

Note. Dependent variable is identified regulation, B-unstandardised regression coefficient; R^2-the proportion of the variance of the dependent variable explained by an independent variable; BCa 95% lower and BCa 95% Upper-the borders of the 95% confidence interval after bootstrapping (Bias corrected and accelerated method) based on 1000 samples.

Table S3

Curvilinear regression analysis of external regulation as a criterion and overplacement as a predictor variable

	B	R^2	BCa 95% Lower	BCa 95% Upper

Constant	2,93		2.67	3.12
Overplacement	.04	.01	-.10	.20
Overplacement ²-quadratic term	-.04	.04	-.21	.10

Note. Dependent variable is external regulation; B-unstandardised regression coefficient; R^2-the proportion of the variance of the dependent variable explained by an independent variable; BCa 95% lower and BCa 95% Upper-the borders of the 95% confidence interval after bootstrapping (Bias corrected and accelerated method) based on 1000 samples.

Table S4

Curvilinear regression analysis of amotivation as criterion and overplacement as predictor variable

	B	R^2	BCa 95% Lower	BCa 95% Upper
Constant	1.69		1.38	1.82
Overplacement	-.07		-.28	.10
Overplacement ²-quadratic term	.16 *	.95	.01	.26

Note. Dependent variable is amotivation; B-unstandardised regression coefficient; R^2-the proportion of the variance of the dependent variable explained by an independent variable; BCa 95% lower and BCa 95% Upper-the borders of the 95% confidence interval after bootstrapping (Bias corrected and accelerated method) based on 1000 samples. * -is significant on the $p<0,05$ level; **- is significant at the $p<0,01$ level.

Table S5

Curvilinear regression analysis of intrinsic motivation as criterion and clinical tribalism (nurses' self-assessment vs.nurses' perception of physicians' competencies) as predictor variable

	B	R^2	BCa 95% Lower	BCa 95% Upper
Constant	3.19		2.74	3.76
Clinical trialism nurses vs. physicians	.31	.17	-0.02	.43
Clinical tribalism²-quadratic term	-.50	.34	-1.08	.20

nurses vs. physicians				

Note. Dependent variable is intrinsic motivation; nurses vs. physicians- difference between nurses' self-assessment And nurses' perception of physicians' competencies; B-unstandardised regression coefficient; R^2-a proportion of the variance of the dependent variable explained by the independent variables; BCa 95% lower and BCa 95% Upper-the borders of the 95% confidence interval after bootstrapping (Bias corrected and accelerated Method) based on 1000 samples. * -is significant on the p<0,05 level; **- is significant on the p<0,01 level.

Table S6

Curvilinear regression analysis of identified regulation as criterion variable and Clinical tribalism (nurses' self-assessment vs.nurses' perception of physicians' competencies) as predictor variable

	B	R^2	BCa 95% Lower	BCa 95% Upper
Constant	3.65		3.28	4.07
Clinical tribalism (nurses vs. physicians)	.37	.09	-.05	.67
Clinical tribalism (nurses vs. physicians)² - quadratic term	-.43	.16	-.83	0.08

Note. Dependent variable is identified regulation; nurses vs. physicians- difference between nurses' self-assessment and nurses' perception of physicians' competencies; B-unstandardised regression coefficient; R^2-a proportion of the variance of the dependent variable explained by the independent variables; BCa 95% lower and BCa 95% Upper-the borders of the 95% confidence interval after bootstrapping (Bias corrected and accelerated Method) based on 1000 samples. * -is significant at the p<0,05 level; **- is significant at the p<0,01 level.

Table S7

Curvilinear regression analysis of external regulation as criterion variable and Clinical tribalism (nurses' self-assessment vs.nurses' perception of physicians' competencies) as predictor variable

	B	R^2	BCa 95% Lower	BCa 95% Upper
Constant	3.21		2.63	5.26
Clinical tribalism(nurses vs.physicians)	-.04	.08	-.31	.05
Clinical tribalism(nurses vs.physicians)² - quadratic term	-.02	.09	-.16	.50

Note. Dependent variable is external regulation; nurses vs. physicians- difference between nurses' self-assessment and nurses' perception of physicians' competencies; B-unstandardised regression coefficient; R^2-a proportion of the

variance of the dependent variable explained by the independent variables; BCa 95% lower and BCa 95% Upper-the borders of the 95% confidence interval after bootstrapping (Bias corrected and accelerated Method) based on 1000 samples. * -is significant on the p<0,05 level; **- is significant at the p<0,01 level.

Table S8

Curvilinear regression analysis of amotivation as criterion and Clinical tribalism (nurses' self-assessment-nurses' perception of physicians' competencies) as predictor variable

	B	R^2	BCa 95% Lower	BCa 95% Upper
Constant	1.77		1.03	2.10
Clinical tribalism(nurses vs.physicians)	-.49**	.01	-.60	.38
Clinical tribalism(nurses vs.physicians)² - quadratic term	.13**	.13	.007	.20

Note. Dependent variable is amotivation; nurses vs. physicians- difference between nurses' self-assessment and nurses' perception of physicians' competencies)B-unstandardised regression coefficient; R^2-a proportion of the variance of the dependent variable explained by the independent variables; BCa 95% lower and BCa 95% Upper-the borders of the 95% confidence interval after bootstrapping (Bias corrected and accelerated Method) based on 1000 samples. * -is significant on the p<0,05 level; **- is significant at the p<0,01 level.

Table S9

Curvilinear regression analysis of intrinsic motivation as criterion and Clinical tribalism (nurses' self-assessment-nurses' perception of paramedics' competencies) as predictor variable

	B	R^2	BCa 95% Lower	BCa 95% Upper
Constant	3.19		2.71	3.77
Clinical tribalism(nurses vs.paramedics)	.31	.02	-.11	.46
Clinical tribalism(nurses vs.paramedics)²-quadratic term	-.11	.11	-.23	.06

Note. Dependent variable is intrinsic motivation B-unstandardised regression coefficient; Clinical tribalism (nurses vs. paramedics)- difference between nurses' self-assessment and nurses' perception of paramedics' competencies); R^2-the proportion of the variance for a dependent variable explained by an independent variable; BCa 95% lower and BCa 95% Upper-the borders of the 95% confidence interval after bootstrapping (Bias corrected and accelerated Method) based on 1000 samples. * -is significant at the p<0,05 level; **- is significant at the p<0,01 level.

Table S10

Curvilinear regression analysis of identified motivation criterion and

Clinical tribalism (nurses' self-assessment-nurses' perception of paramedics' competencies) as predictor variable

	B	R^2	BCa 95% Lower	BCa 95% Upper
Constant	3.63		2.46	4.64
Clinical tribalism(nurses vs.paramedics)	.19	.002	-1.03	1.24
Clinical tribalism(nurses vs.paramedics)²-quadratic term	-.03	.008	-.35	.39

Note. Dependent variable is identified regulation; B-unstandardised regression coefficient; Clinical tribalism (nurses vs. paramedics)-nurses' self-assessment-nurses' perception of paramedics' competencies; R^2-the proportion of the variance for a dependent variable explained by an independent variable; BCa 95% lower and BCa 95% Upper-the borders of the 95% confidence interval after bootstrapping (Bias corrected and accelerated Method) based on 1000 samples. * -is significant at the p<0,05 level; **- is significant at the p<0,01 level.

Table S11

Curvilinear regression analysis of external motivation as the criterion and Clinical tribalism (nurses' self-assessment-nurses' perception of paramedics' competencies) as predictor variable

	B	R^2	BCa 95% Lower	BCa 95% Upper
Constant	3.74		2.91	4.65
Clinical tribalism(nurses vs.paramedics)	-.58	.001	-1.90	.88
Clinical tribalism(nurses vs.paramedics)²-quadratic term	.95	.05	-.25	.34

Note. Dependent variable is external motivation B-unstandardised regression coefficient; R^2-the proportion of the variance for a dependent variable explained by an independent variable; BCa 95% lower and BCa 95% Upper-the borders of the 95% confidence interval after bootstrapping (Bias corrected and accelerated Method) based on 1000 samples. * -is significant at the p<0,05 level; **- is significant at the p<0,01 level.

Table S12

Curvilinear regression analysis of amotivation as the criterion variable and Clinical tribalism (nurses' self-assessment-nurses' perception of paramedics' competencies) as predictor variable

	B	R^2	BCa 95% Lower	BCa 95% Upper
Constant	1.07		.40	1.73
Clinical tribalism(nurses vs.paramedics)	.61	.002	-.61	2.01
Clinical tribalism(nurses vs.paramedics)²-quadratic term	-.14	.03	-.47	.12

Note. Dependent variable is amotivation B-unstandardised regression coefficient; R^2-the proportion

of the variance for a dependent variable explained by an independent variable; BCa 95% lower and BCa 95% Upper-the borders of the 95% confidence interval after bootstrapping (Bias corrected and accelerated Method) based on 1000 samples. * -is significant at the $p<0,05$ level; **- is significant at the $p<0,01$ level

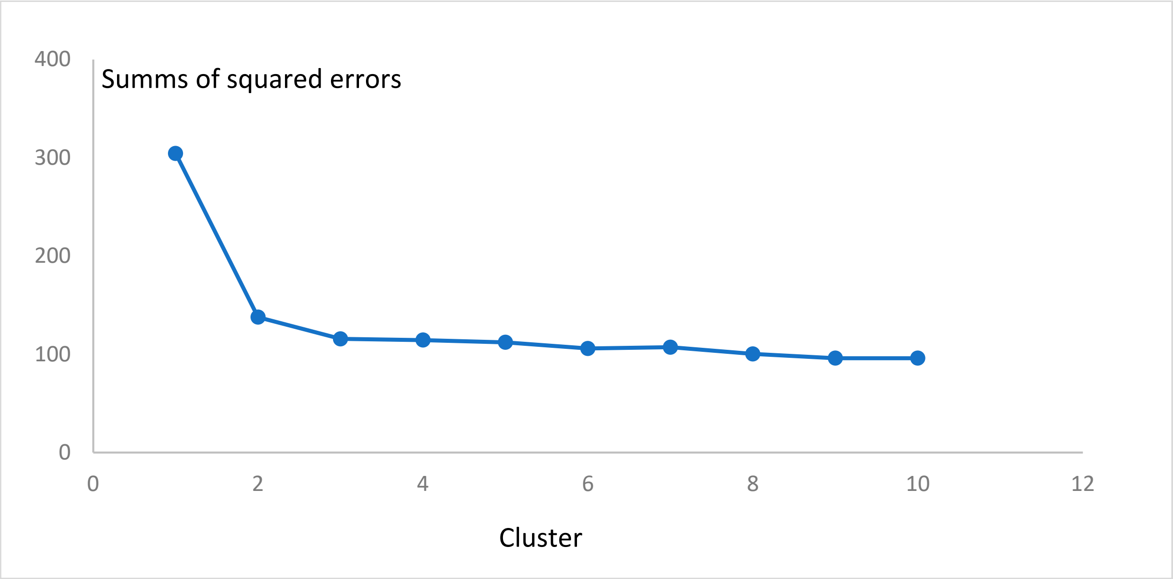

Figure S1. Plot of sums of squared errors against the number of clusters

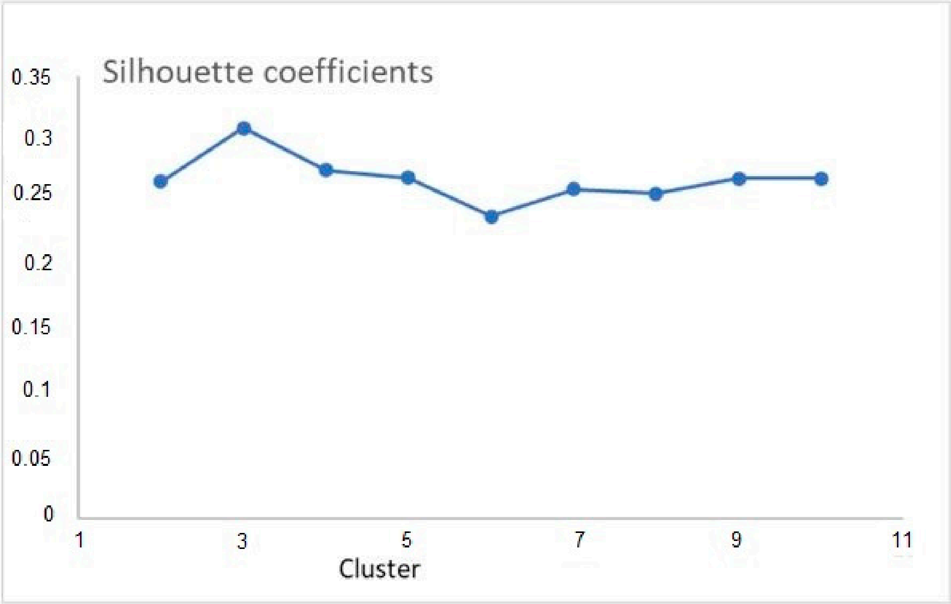

Figure S2. Plot of the means of the Silhouette values against the number of clusters

Table S13

Power analysis for F tests - ANOVA: Fixed effects, omnibus, one-way for the large effect size

	Parameter	Value
Input	Effect size f	0.4
	α err prob	0.05
	Power (1-β err prob)	0.80
	Number of groups	3
Output	Noncentrality parameter λ	10.5600000
	Critical F	3.1428085
	Numerator df	2

Denominator df	63
Total sample size	66
Actual power	0.8180744

Note. Input-previous specified parameter; Output-calculated parameter ;ANOVA-Analysis of Varince

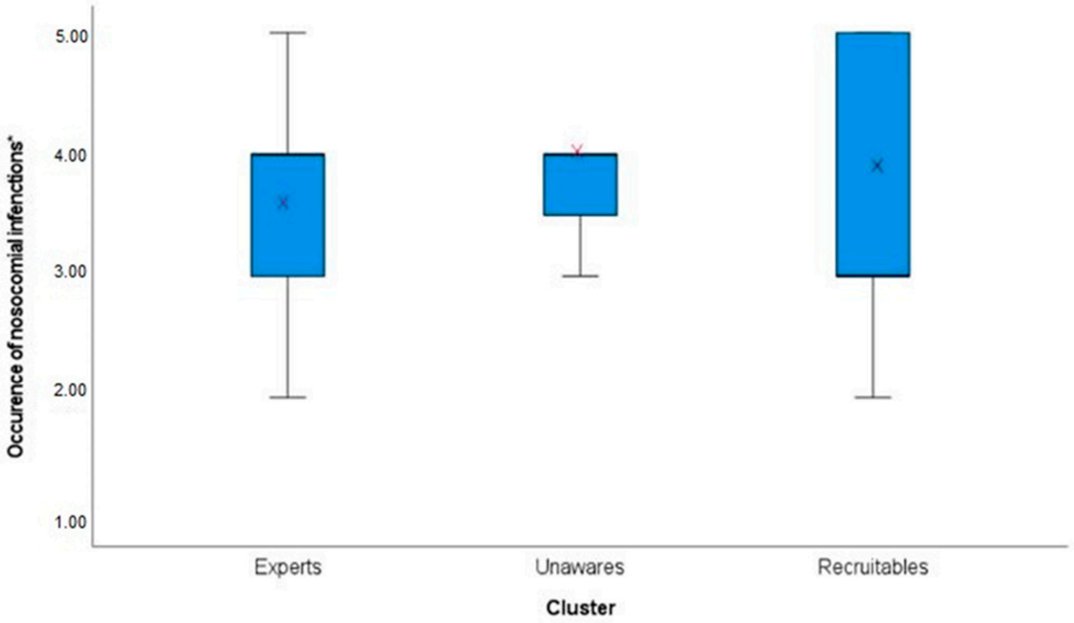

Figure S3. Comparison between the clusters with regards to the mean values of the variable related to patients' Safety (Occurrence of nosocomial infections)

Note. Occurrence of nosocomial infections *- The Item belongs to patients' safety according to international standards of ISO 31000 of medical failure mode effect analysis (FMEA): "How often is it that a patient gets harmed in your working environment? Uncommon (1x >3 years) (1), seldom (once per 3 years) (2), moderate (once per year) (3), often (once per 3 months) (4), very often (once per month) (5)". X-the mean value of the variable in the cluster; One-way ANOVA determined no statistically significant difference between groups.

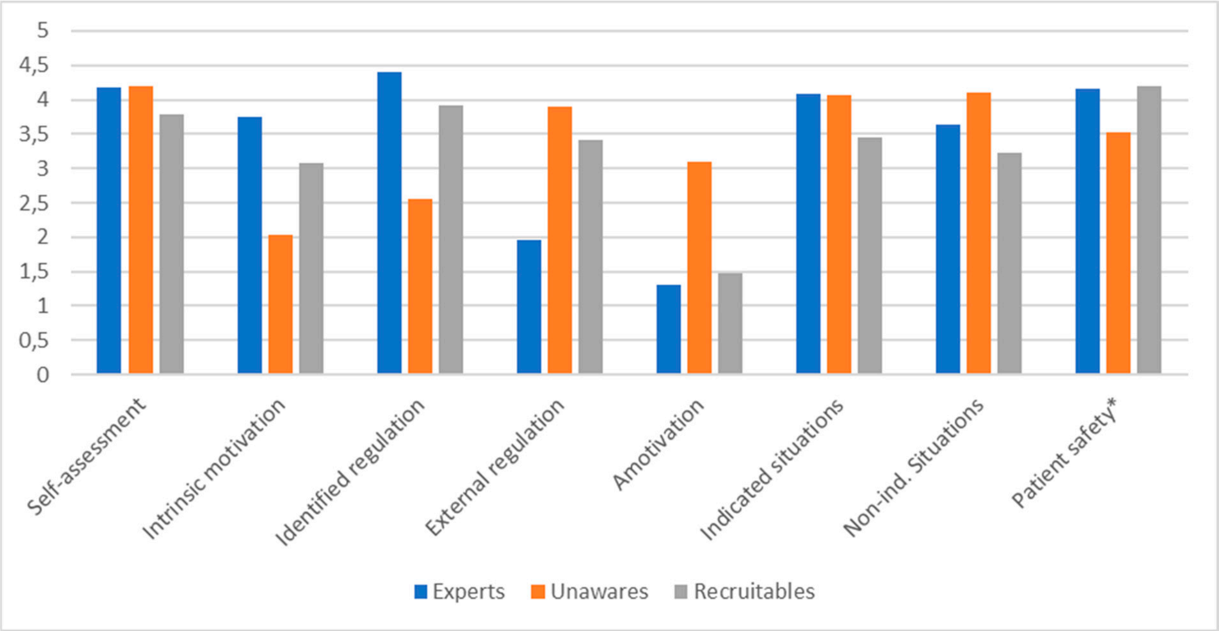

Figure S4. Comparison of the clusters with regards to the mean values of the variables

Note. Patient safety* is the item "The credible maximum effect of omitted hand hygiene is without consequence (1), minor-without any long-lasting effect (2), severe- with longer hospital stay (3), critical -with long-lasting effect (4) and lethal (5) ".
